# High-precision angle adaptive control simulation of synchronous motor for automatic lifting and boarding equipment of aircraft platform

**DOI:** 10.1038/s41598-023-30742-5

**Published:** 2023-03-02

**Authors:** Zhuo Zhang

**Affiliations:** AVIC Xi’an Aircraft Industry Group Co., Ltd, Design Institute, Xi’an, 710089 Shaanxi China

**Keywords:** Engineering, Mathematics and computing

## Abstract

In order to improve the accuracy and adaptability of the Angle control of the aircraft platform automatic lifting and boarding synchronous motors, the high precision Angle adaptive control method of the aircraft platform automatic lifting and boarding synchronous motors is studied. The structure and function of lifting mechanism in automatic lifting and boarding device of aircraft platform are analyzed. The mathematical equation of synchronous motor in automatic lifting and boarding device is established in a coordinate system, the ideal transmission ratio of synchronous motor angle is calculated, and the PID control law is designed according to the transmission ratio. Finally, the high precision Angle adaptive control of the synchronous motor of the aircraft platform automatic lifting and boarding device is realized by using the control rate. The simulation results show that the proposed method can quickly and accurately realize the angular position control of the research object, and the control error is within ± 0.15rd, which has high adaptability.

## Introduction

The automatic landing and boarding equipment of aircraft platform is an airport facility connecting the aircraft and the waiting room to facilitate passengers to enter and leave the cabin. It plays an important role in fast, improving passengers' boarding experience and reducing airport labor costs^1^. The lifting system in the automatic lifting and boarding equipment of aircraft platform is related to the safety of passengers and automatic lifting and boarding equipment of aircraft platform^2^. It is a key part of automatic lifting and boarding equipment of aircraft platform. It is an equipment with high requirements for safety and practicability^3^. Once the automatic lifting and boarding equipment of the aircraft platform has problems, it will cause the elevator and adjustment plate to appear undesired vibration, and even cause the aircraft to lose control.. The lifting system in the automatic lifting and boarding equipment of aircraft platform is controlled by synchronous motor^4^. The lifting system in the automatic lifting and boarding equipment of aircraft platform is controlled by controlling the angle of synchronous motor to realize the application function of automatic lifting and boarding equipment of aircraft platform.

In the mechanical field, synchronous motor has been widely used in industry, national defense, manufacturing and other fields due to its advantages of high power density, small volume and high operation efficiency^5^. The angle positioning can quickly determine the speed and position of rotor^6^. The high-precision angle positioning of synchronous motor has the advantages of high efficiency and fast dynamic response. With the development of synchronous motor, synchronous motor is widely used, which can be used in some blowers, high-power compressors and other devices, which can save mechanical speed-up devices. High-precision angle positioning of synchronous motor can not only improve operation efficiency, but also save energy^7^, so the high-precision angle adaptive positioning method of synchronous motor has become a research hotspot.

Daryabeigi and Mirzaei studied the enhanced emotion and speed deviation control method of synchronous reluctance motor driver^8^. The controller was designed based on the emotion learning and decision-making mechanism in the brain through emotion clues and sensory input, which can accurately track the speed and d-axis stator current reference, but this control method leads to poor operation stability of synchronous reluctance motor. The error of reverse operation is large.Petkar and Kumar et al. studied the predictive control method driven by three-level open winding permanent magnet synchronous motor based on computational effective model^9^. For the open winding permanent magnet synchronous motor powered by three-level inverter, it will produce small torque and stator flux fluctuation. However, the calculation time required for predictive control variables is very long. The maximum four voltage vectors are used to replace the 19 voltage vectors in the traditional three-level model to predict the current control, which reduces the number of predictions and the calculation time of predictions. However, this method has poor connectivity between electronic signals and mechanical models, so it is difficult to ensure that the computer simulation results match the actual data. Tornello and Scarcella studied the combined method of rotor position estimation and temperature monitoring in sensorless synchronous reluctance motor drive^10^, estimated the rotor position of synchronous motor by processing the voltage induced in thermistor box, and directly used this method to build sensorless controller or improve the performance of traditional model-based sensorless control system. The disadvantage of this method is that the calculation intensity is very large, high computing equipment needs to be configured, and the operation speed and the response speed of the algorithm are very slow.

In view of the problems existing in the above literature, a high-precision Angle adaptive control simulation method was proposed for the synchronous motor of aircraft platform automatic lifting and boarding equipment, the structure of aircraft platform automatic lifting and boarding equipment was designed, the shaft components of the synchronous motor in the automatic lifting and boarding equipment of aircraft platform were analyzed, and the Angle modeling of the synchronous motor in the automatic lifting and boarding equipment of aircraft platform was completed. In this paper, the ideal transmission ratio of synchronous motor angle of automatic platform lifting and boarding device is calculated, and the association rule algorithm and PID technology are introduced into it. The PID control law is used to control the synchronous motor of aircraft platform automatic lifting and boarding equipment.

## Materials and methods

### Structure of automatic lifting and boarding equipment for aircraft platform

**Figure 1:**
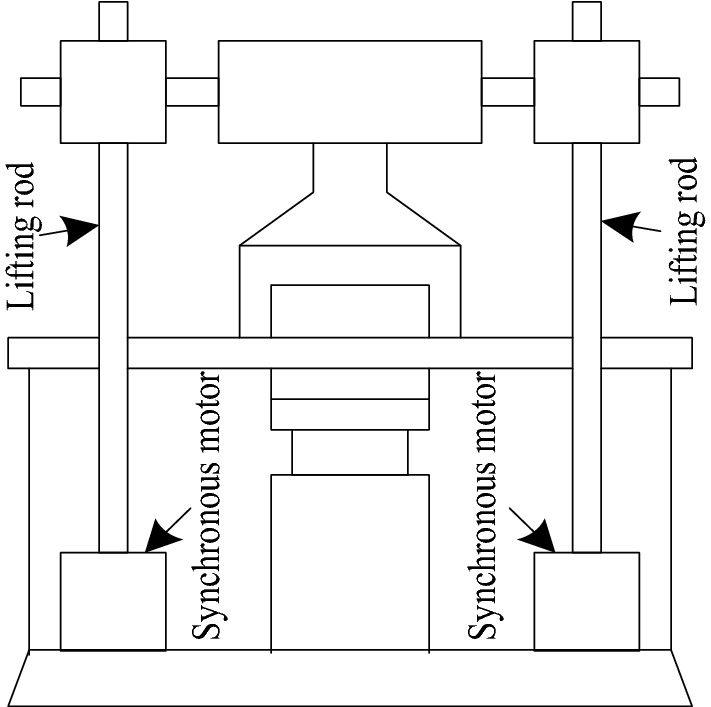
Schematic diagram of lifting mechanism in automatic lifting and boarding equipment of aircraft platform.

In the structure of automatic lifting boarding equipment for aircraft platform, there is a lifting mechanism in the form of gantry structure, and the structural diagram of the lifting mechanism is shown in Fig. 1.

The two lifting rods in the lifting mechanism of the automatic lifting and boarding equipment of aircraft platform are driven by two synchronous motors of the same model. When the automatic lifting and boarding equipment of aircraft platform works, the positions of two lifting rods are required to keep real-time synchronization^11^; the lifting mechanism also applies more than one ton of pressure to the workpiece. At this time, it is still necessary to ensure that the moving position of the lifting mechanism remains synchronous, so as to achieve the purpose of uniform loading. In order to meet the needs of working position initialization and equipment calibration of automatic lifting and boarding equipment of aircraft platform, the lifting mechanism also needs to have synchronous zero return function.

### Angle modeling of synchronous motor for automatic lifting and boarding equipment of aircraft platform

The mathematical equation of the motor under the specified coordinates according to the current and inductance of the synchronous motor in the coordinate axis of the automatic lifting and boarding equipment of aircraft platform is calculated. The current of the synchronous motor in the automatic lifting and boarding equipment of the aircraft platform is analyzed by applying the size of the synchronous motor stator in the automatic lifting and boarding equipment of the aircraft platform^12^. The magnetic rotation of synchronous motor is calculated by using the position Angle of synchronous motor in automatic lifting and boarding equipment of aircraft platform. The range of position angle of synchronous motor is analyzed when it rotates clockwise and counterclockwise in automatic lifting and boarding equipment of aircraft platform^13^, to apply the current vector to the stator of the synchronous motor in the automatic lifting and boarding equipment of aircraft platform, analyze the shaft component of the synchronous motor in the automatic lifting and boarding equipment of aircraft platform, and complete the modeling of the angle of the synchronous motor in the automatic lifting and boarding equipment of aircraft platform.

The mathematical equation of synchronous motor in automatic lifting and boarding equipment of aircraft platform in $\left( {d,q} \right)$ coordinate is:

1 $$\left\{ \begin{gathered} \lambda_{0} = \lambda_{d} { - }L^{\prime}_{d} j_{d} \hfill \\ j_{q} = \frac{{L^{\prime}_{q} }}{{\lambda_{q} }} \hfill \\ Z_{e} = 1.5 \cdot l \cdot j_{d} \left( {\lambda_{0} + j_{q} L^{\prime}_{d} - j_{q} L^{\prime}_{q} } \right) \hfill \\ Z^{\prime\prime} = B \cdot l \cdot s + j_{d} j_{q} \hfill \\ \end{gathered} \right.$$

In Eq. (1), $\lambda_{d}$ and $\lambda_{q}$ represent the flux linkage of the synchronous motor in the automatic lifting and boarding equipment of aircraft platform under the coordinate $\left( {d,q} \right)$, $Z_{e}$ represents the magnetic torque of the synchronous motor in the automatic lifting and boarding equipment of aircraft platform, $j_{d}$ and $j_{q}$ represent the current of the synchronous motor in the automatic lifting and boarding equipment of aircraft platform in the coordinates $d$ and $q$, and $L^{\prime}_{d}$ and $L^{\prime}_{q}$ represent the inductance of synchronous motor in automatic lifting and boarding equipment of aircraft platform in $d$ and $q$ axes respectively, $\lambda_{0}$ represents constant, $l$ represents magnetic number of synchronous motor in automatic lifting and boarding equipment of aircraft platform, $A$ represents damping coefficient of synchronous motor in automatic lifting and boarding equipment of aircraft platform, and $s$ represents speed of synchronous motor in automatic lifting and boarding equipment of aircraft platform,$B$ represents rated capacity of synchronous motor in aircraft platform automatic lifting device. When the inductance of the synchronous motor in the automatic lifting and boarding equipment of the aircraft platform is $L^{\prime}_{d} = L^{\prime}_{q}$, the following expression is given:

2 $$Z_{e} = 1.8 \cdot l \cdot j_{d}$$

When the inductance value is applied to the stator of synchronous motor in the automatic lifting and boarding equipment of aircraft platform, the corresponding direction current is, then $j_{d}$ and $j_{q}$ can be expressed as:

3 $$\left\{ \begin{gathered} j_{d} = j_{s} \cos \theta \hfill \\ j_{q} = j_{s} \sin \theta \hfill \\ \end{gathered} \right.$$

where $j_{s}$ represents the magnitude of the application, $\theta$ represents the difference between $\theta^{\prime}_{c}$ and the position angle $\theta^{\prime}$ of the synchronous motor in the automatic lifting and boarding equipment of the aircraft platform. It can be seen from Eq. (3) and $Z_{e}$ obtains the following expression:

4 $$Z_{e} = 1.8 \cdot l \cdot j_{s} \cdot \lambda_{0} \cdot \sin \theta$$

Since $l$ and $\lambda_{0}$ are fixed, when $\pi > \theta^{\prime}_{c} - \theta^{\prime} > 0$ and $Z_{e} > 0$, the synchronous motor in the automatic lifting and boarding equipment of aircraft platform rotates counterclockwise. When $\pi < \theta^{\prime}_{c} - \theta^{\prime} < 0$ and $Z_{e} < 0$, the synchronous motor in the automatic lifting and boarding equipment of aircraft platform rotates clockwise. When $\theta^{\prime}_{c} - \theta^{\prime} = 0$ or $\theta^{\prime}_{c} - \theta^{\prime} = \pi$, the synchronous motor in the automatic lifting and boarding equipment of aircraft platform does not rotate, showing the direction of the rotation angle of the synchronous motor in the automatic lifting and boarding equipment of aircraft platform, including its position information, and the scale of the rotation angle can be calculated by judging the rotation direction of the synchronous motor in the automatic lifting and boarding equipment of aircraft platform^14^.

Assuming that the rotor magnetic pole of the synchronous motor in the automatic lifting and boarding equipment of aircraft platform is N pole, and the current vector $d$ is applied to its stator in the position angle $\theta^{\prime}$ in the $\left( {d,q} \right)$ coordinate, the axial component of the synchronous motor in the automatic lifting and boarding equipment of aircraft platform is $j_{d} = 0$, and the magnetic field generated by $j_{s}$ interacts with the permanent magnet magnetic field^15^. When $\theta^{\prime} + \pi > \theta^{\prime}_{c} > \theta^{\prime}$, the synchronous motor in the automatic lifting and boarding equipment of aircraft platform rotates counterclockwise; when $\theta^{\prime} - \pi < \theta^{\prime}_{c} < \theta^{\prime}$, the synchronous motor in the automatic lifting and boarding equipment of aircraft platform rotates clockwise. When the rotor reaches 90°, the rotation stops. At this time, the position of the magnetic pole of the synchronous motor in the automatic lifting and boarding equipment of aircraft platform is 90 ℃ different from the current vector position. Combined with the above analysis, the modeling of synchronous motor angle $\vartheta$ in automatic lifting and boarding equipment of aircraft platform is completed. The modeling formula is:

5 $$\theta = Z_{e} \times N + j_{d} \times N + j_{q} \times N$$

Using Eq. (5), the modeling of synchronous motor angle in automatic lifting and boarding equipment of aircraft platform is completed.

### Ideal transmission ratio of synchronous motor angle in automatic lifting and boarding equipment of aircraft platform

Based on the modeling of synchronous motor angle in automatic lifting and boarding equipment of aircraft platform, the ideal transmission ratio of synchronous motor angle in automatic lifting and boarding equipment of aircraft platform is calculated, which can bring association rule algorithm and PID technology into it. The reasonable application of PID technology is the key factor to reasonably adjust the synchronous motor and circuit in the automatic lifting and boarding equipment of aircraft platform^16^, and can realize the real-time monitoring of the specific use of the synchronous motor in the automatic lifting and boarding equipment of aircraft platform under the application mode of this technology, so as to adjust the frequency converter in real time according to the current requirements^17^. It provides basic conditions for the implementation of energy-saving standards and the improvement of overall work efficiency. If the gain angular velocity is set as $H_{k}$, the gain efficiency of the synchronous motor in the automatic lifting and boarding equipment of aircraft platform can be obtained as follows:

6 $$\xi_{i} = H_{k} \cdot \lambda_{n} \cdot A$$

In Eq. (6): $H_{k}$ represents the gain angular velocity in the ideal state during the transmission of the synchronous motor in the automatic lifting and boarding equipment of aircraft platform; $\xi_{i}$ represents the high-precision angular angle of the synchronous motor in the automatic lifting and boarding equipment of aircraft platform; $\lambda_{n}$ represents the lateral swing amplitude of the synchronous motor in the automatic lifting and boarding equipment of aircraft platform at a uniform speed.

By weighting the ratio, we can get:

7 $$\frac{{\xi_{i}^{\prime } }}{{\lambda_{i}^{\prime } }} = \frac{{\frac{{a_{n} }}{{S_{n} }}}}{{\left( {m_{i} \cdot f^{2} } \right)\left( {\frac{{X_{a} { - }Y_{b} }}{{K_{2} }}} \right)}} \cdot A$$

where $a_{n}$ represents the lateral acceleration of the synchronous motor in the automatic lifting and boarding equipment of the aircraft platform at a constant speed; $S_{n}$ refers to the actual distance reached by the synchronous motor in the automatic lifting and boarding equipment of the aircraft platform to walk one circle at a constant speed; $m_{i}$ represents the rotor mass of synchronous motor in the automatic lifting and boarding equipment of aircraft platform; $X_{a}$ represents the speed component of the synchronous motor in the $X$ axis direction in the automatic lifting and boarding equipment of the aircraft platform; $Y_{b}$ represents the speed component of the synchronous motor in the automatic lifting and boarding equipment of the aircraft platform in the $Y$ axis direction; $K_{2}$ represents the steady-state coefficient of synchronous motor in automatic lifting and boarding equipment of aircraft platform, and $f^{2}$ represents the steady-state acceleration gain coefficient of synchronous motor in automatic lifting and boarding equipment of aircraft platform.

It can be seen from Eqs. (6) and (7) that the ideal transmission ratio can be changed by the rotation speed of the synchronous motor in the automatic lifting and boarding equipment of the aircraft platform. If the rotation angle is too sensitive, it is difficult to determine the specific value of the rotation speed during the rotation angle of the synchronous motor in the automatic lifting and boarding equipment of the aircraft platform, so a rational state can be set^9^, the speed $s$ can be set to 0 to obtain:

8 $$I_{\min } = \sum\limits_{i = 1}^{n} {\frac{{\frac{{a_{n} }}{{S_{n} }}}}{{\left( {m_{i} \cdot f^{2} } \right)\left( {\frac{{X_{a} { - }Y_{b} }}{{K_{2} }}} \right)}} \cdot A}$$

where $I_{\min }$ represents the value of ideal transmission ratio when the angular speed of synchronous motor in automatic lifting and boarding equipment of aircraft platform is 0; $\xi_{i\max }$ represents the angular speed under critical state when the synchronous motor in the automatic lifting and boarding equipment of aircraft platform runs to the maximum value; $\lambda_{i\max }$ represents the angular movement distance under critical state when the synchronous motor in the automatic lifting and boarding equipment of aircraft platform runs to the maximum value. Thus, the ideal transmission ratio of synchronous motor in automatic lifting and boarding equipment of aircraft platform can be obtained.

### Design of control law

**Figure 2:**
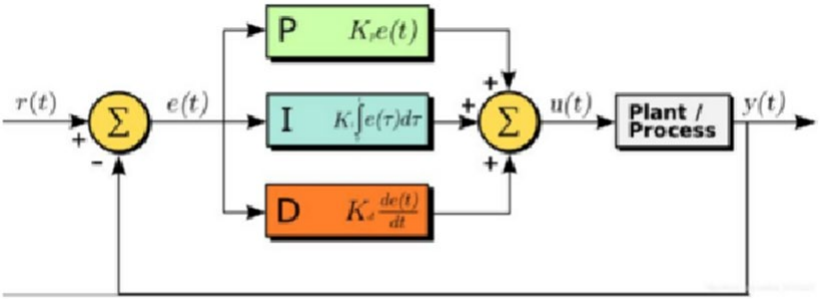
PID control law control structure diagram.

Based on the above transmission ratio, PID control law is adopted for high-precision angle adaptive control of synchronous motor in the automatic lifting and boarding equipment of aircraft platform.Its structure diagram is shown in Fig. 2.

Because the linear PID gain scheduling control law has poor control performance and cannot ensure global stability^18^. Considering that the synchronous motor model in the automatic lifting and boarding equipment of aircraft platform represented by Eq. (1) conforms to the characteristics of strict feedback system, the recursive control law is directly designed for the nonlinear model of synchronous motor in the automatic lifting and boarding equipment of aircraft platform by using the backstepping method, which can not only effectively reduce the design difficulty, but also ensure the global stability of the system. In the backstepping design, the integration of angle error is introduced to improve the angle control accuracy of synchronous motor in automatic lifting and boarding equipment of aircraft platform; an exponential sliding mode reaching law for $d$ and $q$-axis current error is constructed to improve the convergence speed of current error and enhance the robustness of synchronous motor angle adaptive control in automatic lifting and boarding equipment of aircraft platform.

The rotational speed $s$ of the synchronous motor in the automatic lifting and boarding equipment of aircraft platform, the currents $j_{d}^{{}}$ and $j_{q}^{{}}$ of axis $d$ and axis $q$ are selected as the virtual control quantities, and the voltages $u_{d}^{{}}$ and $u_{q}^{{}}$ of axis $d$ and axis $q$ are selected as the actual control quantities. The control goal is that the position tracking error of the synchronous motor in the automatic lifting and boarding equipment of aircraft platform tends to zero:

9 $$\mathop {\lim }\limits_{t \to \infty } \left( {\vartheta \times I - \vartheta^{ * } \times I} \right) = 0$$

where $\vartheta$ and $\vartheta^{ * }$ are the actual rotation angle and the expected reference rotation angle respectively.

In order to obtain the maximum torque output, the synchronous motor in the automatic lifting and boarding equipment of aircraft platform usually adopts vector control^19^. The simplest and effective way is to make:

10 $$j_{d}^{ * } = 0$$

where $j_{d}^{ * }$ is the desired d-axis reference current.

At this time, the electromagnetic torque output by the synchronous motor in the automatic lifting and boarding equipment of aircraft platform can be decoupled as follows:

11 $$Z_{e} = \frac{{1.8 \cdot l \cdot j_{s} \cdot \lambda_{0} \cdot \sin \theta }}{2}$$

The tracking error of synchronous motor angle, speed and $d$, $q$ axis current subsystem in automatic lifting and boarding equipment of aircraft platform is defined as:

12 $$\left\{ \begin{gathered} e_{1} = \vartheta - \vartheta^{ * } \hfill \\ e_{2} = w - w^{ * } \hfill \\ e_{3} = j_{q} - j_{q}^{ * } \hfill \\ e_{4} = j_{d} - j_{d}^{ * } \hfill \\ \end{gathered} \right.$$

where $\vartheta^{ * }$, $w^{ * }$, $j_{q}^{ * }$ and $j_{d}^{ * }$ respectively represent the expected control quantity of each subsystem.

The design steps of backstepping control law are as follows:

1. The formula (1) is regarded as a subsystem, the physical quantity defined in formula (3) is regarded as the state variable of the system, and other quantities are regarded as coefficients. According to the backstepping control method, the error variable is defined:

13 $$\left\{ \begin{gathered} z_{1} = x_{1} - x_{1d} \hfill \\ z_{2} = x_{2} - \alpha_{1} \hfill \\ z_{3} = x_{3} - \alpha_{2} \hfill \\ \end{gathered} \right.$$

where $x_{1d}$ is the expected value of state variable $x_{1}$, $x_{2}$ and $x_{3}$ are virtual control, $\alpha_{1}$ and $\alpha_{2}$ are the stability functions that make the first subsystem stable, and they are also unknown quantities that need to be determined in the next step. In order to avoid over parameterization in adaptive control and singularity of control law, the following variables are defined:

14 $$\left\{ \begin{gathered} F = \frac{A}{J} \hfill \\ \Gamma = \frac{{T_{L} }}{J} \hfill \\ \end{gathered} \right.$$

The estimation error of physical quantity is defined as:

15 $$\left\{ \begin{gathered} \tilde{J} = \hat{J} - J \hfill \\ \tilde{F} = \hat{F} - F \hfill \\ \tilde{\Gamma } = \hat{\Gamma } - \Gamma \hfill \\ \end{gathered} \right.$$

In order to make the first subsystem stable, the uncertainties of moment of inertia $J$, damping coefficient $A$ and load torque $T_{L}$ of the motor system are considered at the same time. The design stability function is:

16 $$\left\{ \begin{gathered} \alpha_{2} = 0 \hfill \\ \alpha_{1} = \frac{{2\hat{J}\left[ { - c_{1} z_{1} + \hat{F}\left( {z_{1} + x_{1d} } \right) + \hat{\Gamma } + \dot{x}_{1d} } \right]}}{{1.8 \cdot l \cdot j_{s} \cdot \lambda_{0} \cdot \sin \theta }} \hfill \\ \end{gathered} \right.$$

2. The stability functions $\alpha_{1}$ and $\alpha_{2}$ defined in the first step ensure the stability of the system, but these two stability functions are the expected values of quadrature axis current and direct axis current respectively, which are not actual physical quantities. The control quantities are designed below to make the quadrature axis current and direct axis current follow their expected values, so as to ensure the stability of the whole system.

The quadrature axis voltage $u_{q}$ is taken as:

17 $$\begin{aligned} u_{q} =& Rj_{q} + PwL_{d} j_{d} + L_{q} \left\{ { + \frac{{2\dot{\hat{J}}}}{{1.8 \cdot l \cdot j_{s} \cdot \lambda_{0} \cdot \sin \theta }}\left[ { + \hat{F}\left( {z_{1} + x_{1d} } \right) + \hat{\Gamma } + \dot{x}_{1d} } \right]} \right. \hfill \\ &+ \frac{{2\hat{J}}}{{1.8 \cdot l \cdot j_{s} \cdot \lambda_{0} \cdot \sin \theta }}\left( {\dot{\hat{F}}x_{1} + \dot{\hat{\Gamma }} + \ddot{x}_{1d} + c_{1} \dot{x}_{1d} } \right) + \left( {\hat{F} - c_{1} } \right) \hfill \\ &\left. {j_{q} - \frac{{2\hat{J}}}{{1.8 \cdot l \cdot j_{s} \cdot \lambda_{0} \cdot \sin \theta }}\left( {\hat{F} - c_{1} } \right)\left( {\hat{F}w + \hat{\Gamma }} \right)} \right\} \hfill \\ \end{aligned}$$

The shaft voltage $u_{d}$ of another actual control quantity is taken as:

18 $$u_{d} = Rj_{d} - PwL_{q} j_{q} - \frac{{2\hat{J}}}{{1.8 \cdot l \cdot j_{s} \cdot \lambda_{0} \cdot \sin \theta }}\left( {L_{d} - L_{q} } \right)L_{d} j_{q} z_{1} - L_{d}$$

The mechanical uncertain parameters are estimated by the following adaptive law:

19 $$\left\{ \begin{gathered} \tilde{J} = \gamma_{1} \left\{ {\hat{F}z_{1} x_{1} - \hat{\Gamma }z_{1} - \dot{x}_{1d} + \left( {\hat{F} - c_{1} } \right)\left[ {1 + \left( {L_{d} - L_{q} } \right)j_{d} } \right]j_{q} } \right\} \hfill \\ \tilde{F} = \gamma_{2} \left[ { - z_{1} x_{1} + \frac{{2\hat{J}}}{{1.8 \cdot l \cdot j_{s} \cdot \lambda_{0} \cdot \sin \theta }}\left( {\hat{F} - c_{1} } \right)wz_{2} } \right] \hfill \\ \tilde{\Gamma } = \gamma_{3} \left[ { - z_{1} + \frac{{2\hat{J}}}{{1.8 \cdot l \cdot j_{s} \cdot \lambda_{0} \cdot \sin \theta }}\left( {\hat{F} - c_{1} } \right)z_{2} } \right] \hfill \\ \end{gathered} \right.$$

In order to ensure the stability of the whole system and consider the influence of parameter uncertainty, the following Lyapunov function is selected:

20 $$\phi_{2} = V_{1} + \frac{{z_{2}^{2} }}{2} + \frac{{z_{3}^{2} }}{2} + \frac{{\tilde{J}^{2} }}{{2\gamma_{1} J}} + \frac{{\tilde{F}^{2} }}{{2\gamma_{2} }} + \frac{{\tilde{\Gamma }^{2} }}{{2\gamma_{3} }}$$

To find the derivative of Lyapunov function can obtain the following equation:

21 $$\dot{\phi }_{2} = \left( {c_{1} z_{1}^{2} } \right) + \left( {c_{2} z_{2}^{2} } \right) + \left( {c_{3} z_{3}^{2} } \right) + \left( {\frac{{1.8 \cdot l \cdot j_{s} \cdot \lambda_{0} \cdot \sin \theta z_{1} z_{2} }}{2J}} \right)$$

where $\theta$ is motor angle, $\lambda_{0}$ is stable equilibrium point, $\sin \theta z_{1} z_{2}$ is state feedback function.

## Results

**Figure 3:**
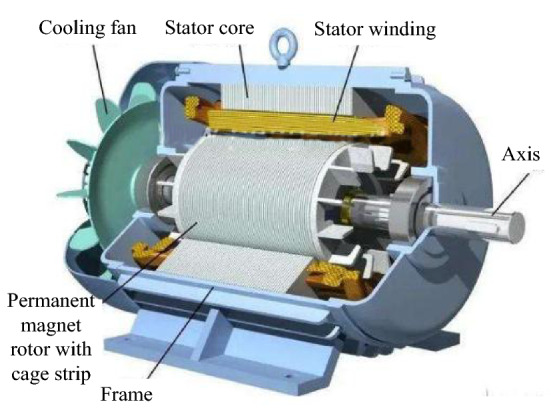
Structure of synchronous motor.

In order to verify the application performance of the high-precision angle adaptive control method for synchronous motor of the automatic lifting and boarding equipment of aircraft platform studied in this paper in the actual synchronous motor high-precision angle control process, taking the synchronous motor used by an automatic lifting and boarding equipment of aircraft platform as the experimental object, its main function is to provide power to the automatic lifting and boarding equipment of aircraft platform. Figure 3 shows the sampling structure of the experimental object. The rotation angle of the experimental object is controlled by the method in this paper, and the control performance of this method is simulated and tested. The results are as follows. At the same time, in order to further illustrate the control performance advantages of the method in this paper, the enhanced emotion and speed deviation control method of synchronous reluctance motor driver in reference^8^ and the calculation effective model predictive control method of permanent magnet synchronous motor drive in reference^9^ are used as the comparison method, and the method in this paper and the two comparison methods are compared to verify the performance of the method in this paper.

The simulation settings are as follows. The type of synchronous motor is AC synchronous motor.The physical parameters of the experimental object are: stator resistance is 1.70 Ω, stator inductance is 9.5mh, pole number is 5, flux linkage is 0.18wb, moment of inertia is 0.001 kg m^2^ and damping coefficient is 4.72 × 10^-5^N m s.

### Simulation results of the proposed method

**Figure 4:**
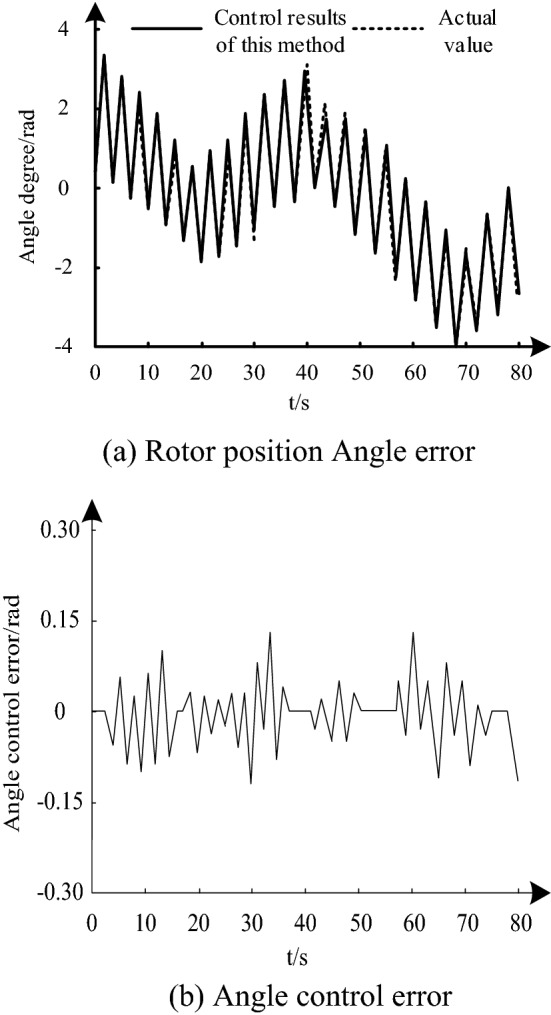
Control results of this method. (**a**) Rotor position Angle error. (**b**) Angle control error.

Using the above simulation parameters and condition settings, under the control of the method in this paper, the angle control effect of the experimental object is shown in Fig. 4a, and the angle control error is shown in Fig. 4b.

It can be seen from Fig. 4a and b that spite of the lack of high order command information and external sudden interference, angular position control can still be realized quickly and accurately under the control of the proposed method. The angular position control error is always within ± 0.15rd. This verifies the applicability of this method.

### Simulation of adaptive control accuracy

PID control law was used to carry out the motor Angle radian adaptive control, the experiment object Angle 10 times. The angle radian of the experimental object is between 9 and 14, which shows that the method in this paper has good adaptive control accuracy for the angle of the experimental object. The experiment is shown in Table 1. At the same time, in order to further illustrate the control performance of the method in this paper, the control results of this method are compared with the control performance of the two comparison methods. The results are shown in Table 1.

**Table 1:** Comparison of motor angle control effect.

Control times	Angle radian		
	Paper method	Enhanced emotional and speed deviation control of synchronous reluctance motor drives	Computationally efficient model predictive control of three-level open-end winding permanent-magnet synchronous motor drive
1	10	7	6
2	9	6	4
3	11	8	8
4	14	4	4
5	9	11	6
6	13	9	3
7	13	6	4
8	10	8	8
9	12	10	6
10	12	14	7

According to the analysis of Table 1, in this method, the radian under the first and second, fifth and eighth angle adaptive control of the experimental object is 9 or 10, and the radian of angle control in the remaining adaptive control tests is between 10 and 14. According to the method of reference^8^, except that the radian under the 5th, 6th, 9th and 10th angle adaptive control reaches 9 or above, the radian of angle control in the remaining adaptive control tests is less than 9; In the adaptive control test, the radian of angle control is less than 9. This shows that the rotation angle control accuracy of the proposed method is significantly higher than that of the two comparison methods.

### Test results of method suitability

When calculating the fitness between the method in this paper and the actual structure, the following formula is required:

22 $$\mu_{H} = \frac{{\sum\limits_{i = 1}^{n} {\left( {a_{i} - a_{0} } \right)\left( {b_{i} - b_{0} } \right)} }}{{\sqrt {\sum\limits_{i = 1}^{n} {\left( {a_{i} - a_{0} } \right)^{2} } \sqrt {\sum\limits_{i = 1}^{n} {\left( {b_{i} - b_{0} } \right)^{2} } } } }}$$

where $\mu_{H}$ is the fitness of the proposedmethod (two comparison methods) for example analysis, and its value range is $\left[ {0,1} \right]$; $a_{i}$ is the fitness of the control method when the scale of the current signal is $i$; $a_{0}$ is the extreme value of the fitness of the control method; $b_{i}$ is the fitness of the control method when the scale of the current signal is $i$; $b_{0}$ is the extreme value of the fitness of the algorithm. The fitness coefficient of the method in this paper is calculated through Eq. (22) (two comparison methods), and the simulation analysis is carried out with the degree of rotation as the variable.

**Figure 5:**
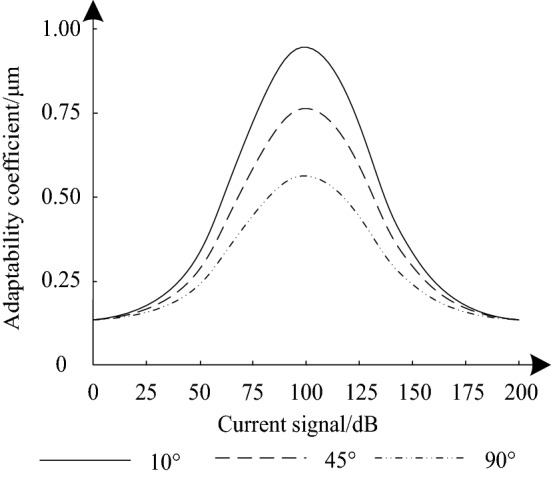
Simulation results of adaptability coefficient.

Using the above designed simulation method, set the degrees of the rotation angle of the experimental object to 10°, 45° and 90°, simulate the control effect of the method in this paper, and output the simulation results through MATLAB software, as shown in Fig. 5.

It can be seen from Fig. 5 that the extreme point of the simulation result for the adaptability coefficient of the method in this paper appears when the current signal is 100 dB, and there are three curves with the angle degree as the variable. When the angle degree is 10°, the extreme value of adaptability coefficient is 0.95 μ m. When the angle degree is 45°, the extreme value of adaptability coefficient is 0.77 μ m. When the rotation angle is 90°, the extreme value of adaptability coefficient is 0.58 μ m. It can be seen that with the increase of angle degree, the adaptability coefficient will decrease accordingly. In order to ensure the accuracy and stability of the test results and avoid the interference of random data, repeat the test for more than 10 times, calculate the average value, and compare it with the adaptability coefficient obtained by the two comparison methods. The results are shown in Table 2.

**Table 2:** Comparison results of average value of adaptability coefficient.

Control times	Comparison results of mean value of adaptability coefficient/μm		
	Paper method	Enhanced emotional and speed deviation control of synchronous reluctance motor drives	Computationally efficient model predictive control of three-level open-end winding permanent-magnet synchronous motor drive
1	0.50/0.94	0.31/0.84	0.26*0.88
2	0.50/0.94	0.30/0.84	0.29/0.88
3	0.49/0.95	0.29/0.85	0.27/0.87
4	0.50/0.91	0.30/0.86	0.28/0.89
5	0.48/0.94	0.27/0.83	0.30/0.90
6	0.50/0.90	0.30/0.82	0.26/0.88
7	0.51/0.89	0.31/0.85	0.28/0.87
8	0.49/0.95	0.28/0.83	0.27/0.86
9	0.50/0.97	0.29/0.86	0.31/0.90
10	0.48/0.98	0.28/0.85	0.27/0.86

Table 2 shows the maximum and minimum values of adaptability coefficient in 10 groups of tests. Through calculation, the average value of the adaptability coefficient of the method in this paper is higher than that of the methods in reference^8^ and^9^. To sum up, the maximum and minimum of the adaptability coefficient in the method of this paper are higher than that of the two comparison methods, which shows that this method is better than the two comparison methods and can have higher accuracy in practice.This is because the mathematical equation of the synchronous motor in the automatic lifting and boarding device of the aircraft platform is established in the d coordinate system, the ideal transmission ratio of the synchronous motor angle in the automatic lifting and boarding device of the aircraft platform is calculated, and the PID control law is designed according to the transmission ratio. Finally, the high precision Angle adaptive control of the synchronous motor of the aircraft platform automatic lifting and boarding device is realized by using the control rate.

## Discussion

Synchronous motor has been widely used in the field of aerospace motion position servo. The basic requirements of its angle tracking control are fast response, no overshoot, no steady-state error and strong robustness. However, the inherent nonlinear characteristics such as multivariable and strong coupling of synchronous motor make it difficult for most conventional control methods to meet the above requirements at the same time. Therefore, the field of synchronous motor control has always been committed to exploring more effective angle tracking control methods. In this paper, the high-precision angle adaptive control method of synchronous motor for aircraft platform’s automatic lifting and boarding equipment is studied, and the control performance of the method is simulated and tested. The results show that this method has good control effect. Angular position control can be realized quickly and accurately. The angular position control error is always within ± 0.15rd. The Angle control accuracy of this method is obviously higher than that of the two comparison methods. The maximum and minimum values of the adaptive coefficient of the proposed method are higher than those of the two comparison methods.The core of this control method is the design of PID control rate. Proportional integral derivative (PID) control is one of the earliest developed control strategies. Because of its simple algorithm, good robustness and high reliability, it is widely used in industrial process control, especially for deterministic control systems that can establish accurate mathematical models. PID controller has a history of nearly 70 years. It has a simple structure, good stability, reliable operation and easy adjustment has become one of the main technologies of industrial control. When the structure and parameters of the controlled object can not be fully mastered, or the accurate mathematical model can not be obtained, and other technologies of control theory are difficult to adopt, the structure and parameters of the system controller must be determined by experience and on-site debugging. At this time, the application of PID control technology is the most convenient. That is, when we do not fully understand a system and the controlled object, or can not obtain the system parameters through effective measurement means, it is most suitable to use PID control technology.

## Conclusion

The high precision Angle adaptive control method of synchronous motor for automatic lifting and boarding device of aircraft platform is studied. Based on the mathematical model of the synchronous motor in the automatic lifting and boarding device of aircraft platform, the PID control law is designed to realize the Angle control of the synchronous motor in the automatic lifting and boarding device of aircraft platform. The simulation results show that the maximum and minimum values of the adaptive coefficient of the proposed method are higher than those of the two comparison methods. The proposed method can realize the high precision Angle control of the automatic lifting of aircraft platform and the synchronous motor of boarding equipment. This method is expected to lay a foundation for further research on permanent magnet synchronous motor. It provides an effective means and tool for analyzing and designing the control strategy of real-time high-performance synchronous motor.This control method can improve the anti-interference performance of permanent magnet synchronous motor and has a good application prospect in the field of aircraft platform automatic lifting and boarding equipment. It can meet the high precision and high reliability requirements of aircraft platform automatic lifting and boarding equipment.

## Data Availability

All relevant data are included in the paper.
